# Long Non-Coding RNA AL139385.1 as a Novel Prognostic Biomarker in Lung Adenocarcinoma

**DOI:** 10.3389/fonc.2022.905871

**Published:** 2022-05-16

**Authors:** Xi Chen, Jishu Guo, Fan Zhou, Wenjun Ren, Xiaobin Huang, Jun Pu, Xiaoqun Niu, Xiulin Jiang

**Affiliations:** ^1^ Department of Neurosurgery, The Second Affiliated Hospital of Kunming Medical University, Kunming, China; ^2^ Institute for Ecological Research and Pollution Control of Plateau Lakes, School of Ecology and Environmental Science, Yunnan University, Kunming, China; ^3^ Hematology and Rheumatology Department, The Pu’er People’s Hospital, Pu’er, China; ^4^ Department of Cardiovascular Surgery, The First People’s Hospital of Yunnan Province, Kunming, China; ^5^ Department of Respiratory Medicine, Second Hospital of Kunming Medical University, Kunming, China; ^6^ Kunming College of Life Science, University of Chinese Academy of Sciences, Beijing, China

**Keywords:** AL139385.1, lung adenocarcinoma, prognosis biomarker, DNA methylation, ceRNA, cell proliferation, cell migration

## Abstract

Lung adenocarcinoma (LUAD) is the most common histological lung cancer, and it is the leading cause of cancer-related deaths worldwide. LncRNA-AL139385.1 (ENSG00000275880) is a novel lncRNA that is abnormally expressed in various cancer types including LUAD. However, the underlying biological function and potential mechanisms of AL139385.1 driving the progression of LUAD remain unclear. In this study, we investigated the role of AL139385.1 in LUAD and found that DNA hypomethylation was positively correlated with AL139385.1 expression in LUAD. Moreover, we uncover that the expression of AL139385.1 in LUAD tissues was significantly higher than that of AL139385.1 expression in adjacent normal tissues. Kaplan–Meier survival analysis showed that patients with higher AL139385.1 expression correlated with adverse overall survival and progression-free survival. Receiver operating characteristic (ROC) curve analysis showed that the area under the curve (AUC) value of AL139385.1 was 0.808. Correlation analysis showed that AL139385.1 expression was associated with immune infiltration in LUAD. We also found that AL139385.1 was upregulated in LUAD cancer tissues and cell lines. Knockdown of AL139385.1 significantly inhibited cell proliferation and migration abilities of LUAD. Finally, we constructed a ceRNA network that includes hsa-miR-532-5p and four mRNAs (GALNT3, CYCS, EIF5A, and ITGB4) specific to AL139385.1 in LUAD. Subsequent Kaplan–Meier survival analysis suggested that polypeptide N-acetylgalactosaminyltransferase 3 (GALNT3), cytochrome c, somatic (CYCS), eukaryotic translation initiation factor 5A (EIF5A), and integrin subunit beta 4 (ITGB4), were potential prognostic biomarkers for patients with LUAD. In conclusion, this finding provides possible mechanisms underlying the abnormal upregulation of AL139385.1 as well as a comprehensive view of the AL139385.1-mediated competing endogenous RNAs (ceRNA) network in LUAD, thereby highlighting its potential role in diagnosis and therapy.

## Introduction

Non–small cell lung cancer (NSCLC) is the main histological type of lung cancer (approximately accounts for 85% of newly diagnosed cases), whereas lung adenocarcinoma (LUAD) is a classification of NSCLC with high morbidity and mortality (1). In the past several decades, considerable progress has been made in surgical treatment, radiotherapy, chemotherapy, molecular targeted therapy, and immunotherapy for lung cancer (2). However, most patients with NSCLC are already in the advanced stage when it is initially diagnosed resulting in a short 5-year overall survival (OS) rate of patients (2). To better treat LUAD, discovering new biomarkers, therapeutic targets, and drugs for effectively preventing the development of lung cancer is urgently needed.

Long non-coding RNAs (lncRNAs) have been functionally annotated with the high-throughput sequencing technologies, which were a class of non-coding RNAs with more than 200 nucleotides in length (3). Studies have demonstrated that lncRNAs play a vital role in epigenetic, cell cycle, and cell differentiation regulation (4). Increasing evidence revealed that lncRNAs are aberrantly regulated in various cancer types and may act crucial roles in tumor progression of cell migration, proliferation, genomic stability, and survival in LUAD (5–7). Owing to lncRNAs are involved in the lung adenocarcinoma (LUAD)-related gene expression and also be regulated by transcription factors in diverse human cancer progression (8). It have been confirmed that lncRNAs to compete with microRNAs (miRNAs) as a molecular sponge result in the degradation of target mRNAs (9). Therefore, the molecular regulation mechanism of tumor-related lncRNAs in LUAD has become the focus of scientific research. Many lncRNAs are involved in the progression of LUADs, such as taurine up-regulated 1 (TUG1), HOX transcript antisense RNA (HOTAIR), Wilms tumor 1 associated protein pseudogene 1 (WTAPP1), and prostate cancer associated transcript 1 (PCAT1). (10–13). A recent study also reported several abnormal expressed lncRNAs in lung cancer, such as lncRNA-XIST and JPX (14, 15). In our previous study, we developed a new method called CVAA (Cross-Value Association Analysis), which functions without a normalization and distribution assumption. We applied it to large-scale pan-cancer transcriptome data generated by The Cancer Genome Atlas (TCGA) project and successfully discovered numerous new differentially expressed genes (DEGs) (16). AL139385.1 is one of these DEGs. However, the clinical significance, prognostic value, diagnostic value, immune infiltration, and potential biological function of AL139385.1 in LUAD remain elusive. Nevertheless, the function, potential mechanism, and prognostic significance of lncRNA-AL139385.1 in LUAD remain unclear. 

In the current study, the expression of AL139385.1 in LUAD was measured and confirmed. The abnormal expression of AL139385.1 was correlated with the clinical outcome of patients with LUAD. Moreover, the correlation between AL139385.1 expression and immune infiltration was analyzed to explore the potential mechanisms involved in AL139385.1 modulation in the progression of LUAD. Meanwhile, CCK8, BrdU, wound healing, and transwell assays were utilized to determine the biological function of AL139385.1 in LUAD progression. Finally, we also conducted data mining on TCGA to identify upstream regulation mechanisms and downstream network of AL139385.1. In summary, our findings indicate the potential role of AL139385.1 in regulating tumor progression and its potential application in the diagnosis and prognostic evaluation in LUAD.

## Materials and Methods

### TCGA Datasets

Download transcription and clinical information of LUAD was downloaded from TCGA (https://portal.gdc.com) (17). RNA-seq gene expression data of workflow type fragments per kilobase of transcript per million fragments mapped (FPKM) were transformed into transcripts per million (TPM) format and log2 transformation for further study. The timeROC analysis was used to compare the predictive accuracy of AL139385.1 gene in LAUD.

### The Human Protein Atlas

The Human Protein Atlas (HPA) (https://proteinatlas.org/) including the normal tissue and tumor tissue protein levels of human gene expression profile information (18). In this study, HPA database utilized to analysis the protein expression of GALNT3, CYCS, EIF5A, and ITGB4 in lung cancer tissues.

### Cox Regression Analysis and Kaplan–Meier Survival Analysis

We utilized cox regression analysis to examine the correlation between AL139385.1 expression and OS of patients with LUAD. The Kaplan–Meier methods employed to determine the prognosis of patients with high and low AL139385.1 expression.

### Gene Set Enrichment Analysis

In the present research, we utilized the linkedomics database (http://www.linkedomics.org/login.php) obtained the co-expression genes of AL139385.1 in LUAD. The gene set kegg.v6.2.symbols.gmt, which served as a reference gene set, was downloaded from the Molecular Signatures Database (MSigDB) (http://software.broadinstitute.org/gsea/msigdb) (19–21).

### Tumor Immune Estimation Resource Database

The Tumor Immune Estimation Resource (TIMER) (https://cistrome.shinyapps.io/timer/) is a comprehensive online database, including the diverse cancer types related to immune infiltrating (22). In this finding, TIMER used to examine the correlation between GALNT3, CYCS, EIF5A, and ITGB4 expression and six types of immune infiltrates (B cells, CD4^+^ T cells, CD8^+^ T cells, neutrophils, macrophages, and dendritic cells) in LUAD.

### Immune Infiltration Analysis by single-sample Gene Set Enrichment Analysis (ssGSEA)

We used a GSVA R package to quantify the LUAD immune infiltration of 24 tumor-infiltrating immune cells in tumor samples through ssGSEA. According to the 509 gene signatures of 24 tumor-infiltrating lymphocytes (TILs), the relative enrichment score of every immunocyte was quantified (23). The correlation between AL139385.1 and infiltration levels of immune cells was analyzed by the Spearman correlation, and these immune cells with the different expression groups of AL139385.1 were analyzed by the Wilcoxon rank sum test.

### The Targets Gene of miR-532-5p Predicted by Various Database

In this study, starBase (https://starbase.sysu.edu.cn/), miRDB (http://mirdb.org/), miRWalk (http://zmf.umm.uni-heidelberg.de/apps), and miRGator (http://mirgator.kobic.re.kr/) (9, 12–14) were utilized to predict the potential targets gene of miR-532-5p. StarBase was also used to analyze the correlation between miR-532-5p expression and AL139385.1

### Cell Culture Conditions

Lung cancer cells lines including human normal bronchial epithelial cell (BEAS-2B) and three human LUAD cells (H1650, H1299, and A549 cells) were purchased from Chinese Academy of Sciences Cell Bank (China) and cultured in RPMI 1640 medium (Corning) including 10% fetal bovine serum (FBS) and 1% penicillin/streptomycin at 37°C an atmosphere containing 95% air and 5% CO_2_.

### Constructs, Transfection, and Infection

Human AL139385.1 full-length cDNA was synthesized (Shanghai Generay Biotech) and sub-cloned into pCDH-CMV-E2F-eGFP lenti-viral vector. Independent small hairpin RNAs (shRNAs) targeting AL139385.1 were synthesized and cloned into the lentiviral plasmid pLKO.1 (Addgene, Cambridge, USA). The lenti-viruses were generated according to the manufacturer’s protocol. The AL139385.1 shRNAs and control scrambled shRNA were transfected into human embryonic kidney cells (HEK)-293T cells with the psPAX2/pMD2.G plasmids (Addgene) using calcium phosphate. After transfection, the cell supernatants were harvested and used to infect H1299 and A549 cells, and the stably lenti-viral infected cells were selected with puromycin. The primer used in this study is as follows: AL139385.1 shRNA#1: GGCCCCGCGCGCGGGCTGCCC, AL139385.1 shRNA#2: GGTCCCAGCTCTGGCCTAAGA.

### Quantitative Real-Time PCR

The qRT-PCR assay was performed as documented (15). The primer sequences are list follows: AL139385.1-F: CGAGAGCAATGCGACAACGAC, AL139385.1-R: CTCCACCTGCCAGCAAAATC; β-actin-F: CTTCGCGGGCGACGAT,β-actin-R: CCATAGGAATCCTTCTGACC. The expression quantification was obtained with the 2^−ΔΔCt^ method.

### CCK 8 and BrdU Incorporation

Cell viability and growth was determined using CCK8 assays in 96-well plates. Cells were transfected with the relevant plasmids culturing for 48 h, followed by incubation with 8 μl of CCK8 for 4 h. Absorbance was read at 450 nm using a spectrophotometer. For BrdU incorporation assay, indicated cells were cultured in eight-well chamber slides for 24 h, Subsequently, indicated cells were fixed with 4% paraformaldehyde (PFA) at room temperature for 20 min and then incubated with BrdU primary antibody (Abcam, ab6326) followed by secondary antibody detection.

### Cell Migration Assay

To produce a wound, the monolayer cells in six-well plate were scraped in a straight line with pipette tips. Plate was then washed with warm phosphate-buffered saline (PBS) to remove detached cells. Photographs of the scratch were taken at indicated time points using Nikon inverted microscope (Ti-S). Gap width was calculated with GraphPad Prism software. For transwell assay, 1 × 10^4^ to 2 × 10^4^ cells in 100 μl of serum-free medium were plated in an 8.0-μm, 24-well plate chamber insert (Corning Life Sciences, catalog no. 3422), with medium containing 10% FBS at the bottom of the insert. Cells were incubated for 24 h and then fixed with 4% PFA for 20 min. After washing, cells were stained with 0.5% crystal violet blue. The positively stained cells were examined under the microscope.

### Statistical Analyses

The significance of the data between two experimental groups was determined by Student’s t-test, and multiple group comparisons were analyzed by one-way ANOVA. P < 0.05 (*), P < 0.01 (**), and P < 0.001 (***) were significant.

## Results

### AL139385.1 Is Highly Expressed in LUAD Tissues

**Figure 1:**
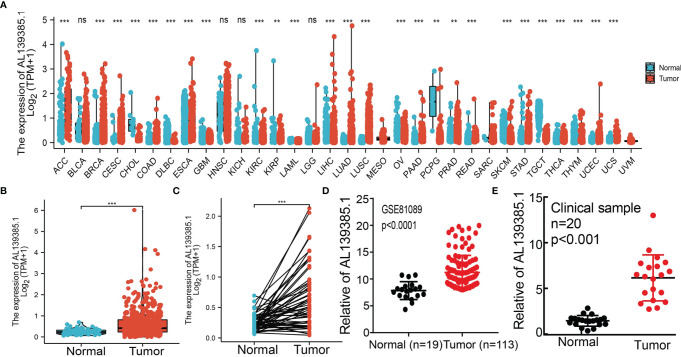
AL139385.1 was overexpressed in LUAD. **(A)** AL139385.1 was highly expressed in 14 of the 33 cancers compared with normal tissue. **(B–D)** AL139385.1 was overexpressed in LUAD examine by TCGA and GEO datasets. **(E)** Relative AL139385.1 expression detected by RT-qPCR in 20 paired lung cancer and noncancerous tissues. ACC, adrenocortical carcinoma; BLCA, bladder urothelial carcinoma; BRCA, breast invasive carcinoma; CESC, cervical squamous cell carcinoma and endocervical adenocarcinoma; CHOL, cholangiocarcinoma; COAD, colon adenocarcinoma; DLBC, lymphoid neoplasm diffuse large B-cell lymphoma; ESCA, esophageal carcinoma; GBM, glioblastoma multiforme; HNSC, head and neck squamous cell carcinoma; KICH, kidney chromophobe; KIRC, kidney renal clear cell carcinoma; KIRP, kidney renal papillary cell carcinoma; LAML, acute myeloid leukemia; LUAD, brain lower grade glioma; LIHC, liver hepatocellular carcinoma; LUAD, lung adenocarcinoma; LUSC, lung squamous cell carcinoma; MESO, mesothelioma; OV, ovarian serous cystadenocarcinoma; PAAD, pancreatic adenocarcinoma; PCPG, pheochromocytoma and paraganglioma; PRAD, prostate adenocarcinoma; READ, rectum adenocarcinoma; SARC, sarcoma; SKCM, skin cutaneous melanoma; STAD, stomach adenocarcinoma; TGCT, testicular germ cell tumors; THCA, thyroid carcinoma; THYM, thymoma; UCEC, uterine corpus endometrial carcinoma; UCS, uterine carcinosarcoma; UVM, uveal melanoma. NS: P > 0.05, **P < 0.01, and ***P < 0.001.

To examine the RNA expression patterns of AL139385.1 in diverse cancer types, we used TCGA and GTEx datasets conducted analysis. On the basis of the best cutoff score, results show that AL139385.1 was upregulated in 14 of the 33 cancers compared with normal tissue **(** **Figure 1A** **)**. To examine AL139385.1 RNA expression in LUAD, we analyzed AL139385.1 expression data in TCGA. We uncover that AL139385.1 was highly expressed in 535 tumor tissues than 59 normal prostate tissues in LUAD **(** **Figure 1B** **)**. Furthermore, we found that AL139385.1 was highly expressed in 59 pairs of LUAD cancer samples than matched adjacent normal samples in TCGA data **(** **Figure 1C** **)**, and similar results were obtained by using the Gene Expression Omnibus (GEO) dataset **(** **Figure 1D** **)**. To further validation of AL139385.1 expression in lung cancer tissues, we conducted qRT-PCR assay to examine AL139385.1 expression in 20 pairs of lung cancer tissues and adjacent non-cancerous tissues and found significantly higher AL139385.1 expression in lung cancer tissues than in adjacent normal tissues **(** **Figure 1E** **)**.

### Overexpression of AL139385.1 Was Associated With Adverse Clinical Parameters in Lung Adenocarcinoma

**Figure 2:**
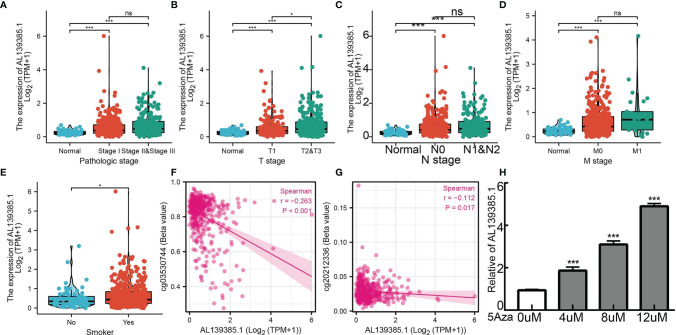
Analysis of the clinical significance and DNA methylation of AL139385.1 in lung adenocarcinoma. Correlation with AL139385.1 expression and clinicopathologic characteristics, including **(A)** pathological stage, **(B–D)** TNM stage and **(E)** smoking. **(F, G)** AL139385.1 expression negatively correlates with mean AL139385.1 promoter methylation levels in TCGA LUAD cohort. **(H)** AL139385.1 expression in A549 cells was significantly upregulated in a dose-dependent manner after 5-azacytidine treatment. NS: P > 0.05, *P < 0.05, and ***P < 0.001.

We employed the TCGA LUAD datasets to examine the clinical relevance of AL139385.1 in LUAD. **As shown in** **Figure 2**, AL139385.1 expression was significantly associated with advanced pathological stage, TNM stage, and age **(** **Figures 2A–E** **)**. A growing body of evidence confirmed that DNA methylation plays a central role in gene expression regulation and cancer progression. Therefore, we decided to investigate the potential association between DNA methylation and AL139385.1 expression. Regression analysis demonstrated a negative correlation between AL139385.1 expression and its diverse DNA methylation sites **(** **Figures 2F, G** **).** To further examine whether hypomethylation can increase AL139385.1 expression, we conducted *in vitro* experiments by adding 5-azacytidine to A549 cells. We confirmed that 5-azacytidine significantly elevate AL139385.1 expression in a dose-dependent manner **(** **Figure 2H** **)**. Together, these results confirmed that DNA methylation may plays a critical role in regulating AL139385.1 expression n LUAD.

### Diagnostic and Prognostic Value

**Figure 3:**
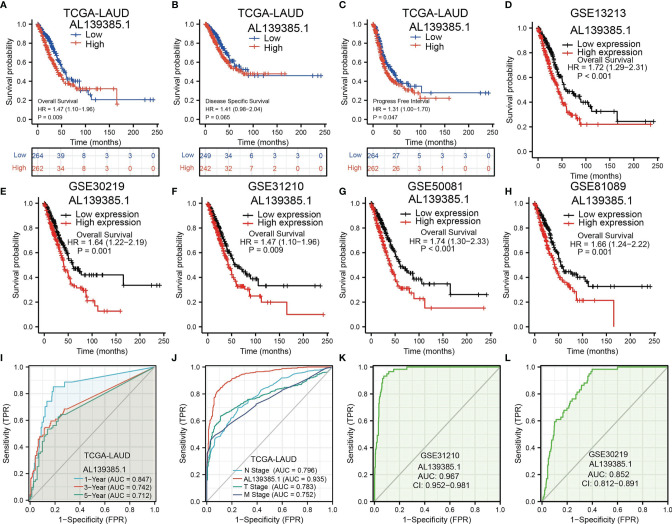
The prognostic and diagnostic value of AL139385.1 expression in LUAD. **(A-C)** Kaplan–Meier survival curves showed that lung adenocarcinoma patients with high AL139385.1 expression exhibited poor overall survival, disease-specific survival, and progression-free survival of AL139385.1 in LUAD determine by TCGA-LUAD dataset. **(D–H)** Survival curves of OS from GEO datasets. **(I)** ROC curves showed that the AUC of AL139385.1 for predicting 1-, 3-, and 5-year survival of patients is 0.847, 0.742, and 0.712, respectively. **(J)** The prediction performance of AL139385.1 is higher than TNM stage. **(K, L)** ROC curves were used to determine the diagnostic value of AL139385.1 in lung adenocarcinoma based on GEO datasets.

Next, to assess the differences in survival time between low and high AL139385.1 expression in patients with LUAD, the Kaplan–Meier method was performed. Kaplan–Meier analysis showed that patients with higher AL139385.1 expression had markedly adverse OS and progression-free survival (PFS) in patients with LUAD. No correlation between disease-free survival (DSS) and AL139385.1 level was observed **(** **Figures 3A–C** **)**. Moreover, we also explore the prognostic value of AL139385.1 in LUAD using various GEO datasets. Results suggested that the OS of high AL139385.1 expression was significantly poorer than low AL139385.1 expression **(** **Figures 3D–H** **)**. Meanwhile, the time-dependent ROC curve was also used to assess the predictive power of the AL139385.1 in predicting 1-, 3-, and 5-year OS, and the AUC for 1-, 3-, and 5-year survival rate of patients with LUAD was 0.847, 0.742, and 0.712 in the TCGA cohort, respectively **(** **Figure 3I** **)**. It is noteworthy that, compared with the clinical common indicators including T stage, N stage, and M stage, AL139385.1 had a higher predictive power in the TCGA cohort **(** **Figure 3J** **)**. We also used GEO dataset to validate the diagnostic value of AL139385.1 in lung cancer. ROC curve results showed that the expression of AL139385.1 in lung cancer was 0.967 (CI 0.952–0.981) and 0.852 (CI 0.812–0.891) **(** **Figures 3K, L** **)**. Together, these results suggest that AL139385.1 is a moderately sensitive index for predicting the prognosis of lung cancer patients and can act as an effective prognostic biomarker in lung cancer.

### Univariate and Multivariate Cox Regression Analyses of Different Parameters on Overall Survival

We performed univariate cox regression analysis in the TCGA-LAUD cohort to determine whether AL139385.1 expression level and pathologic stage might be valuable prognostic biomarkers. Univariate cox regression analysis results show that high expression of AL139385.1, pathologic stage, and TNM stage were associated with OS in patients with LUAD. To ascertain whether AL139385.1 expression level could be an independent prognostic factor for patients with LUAD, multivariate cox regression analysis was performed. We confirmed that upregulation of AL139385.1 was a significant independent prognostic factor in the TCGA-LAUD cohort that directly correlated with adverse clinical outcomes, along with pathological stage and T stage **(** **Table 1** **)**.

**Table 1:** Univariate and multivariate Cox regression analyses of different parameters on overall survival in lung adenocarcinoma.

Characteristics	Total (N)	Univariate analysis		Multivariate analysis	
		Hazard ratio (95% CI)	P value	Hazard ratio (95% CI)	P value
Pathologic stage	518				
Stage I and Stage II	411				
Stage III and Stage IV	107	2.664 (1.960–3.621)	<0.001	5.702 (2.080–15.637)	<0.001
T stage	504				
T1	175				
T2 and T3	329	1.658 (1.175–2.341)	0.004	1.684 (1.075–2.640)	0.023
N stage	510				
N0 and N1	437				
N2 and N3	73	2.321 (1.631–3.303)	<0.001	0.483 (0.173–1.347)	0.164
M stage	377				
M0	352				
M1	25	2.136 (1.248–3.653)	0.006	0.352 (0.114–1.087)	0.069
Smoker	512				
No	72				
Yes	440	0.894 (0.592–1.348)	0.591		
AL139385 1	526	1.317 (1.087–1.596)	0.005	1.254 (1.004–1.565)	0.046

### Construction and Validation of AL139385.1-Based Nomogram

**Figure 4:**
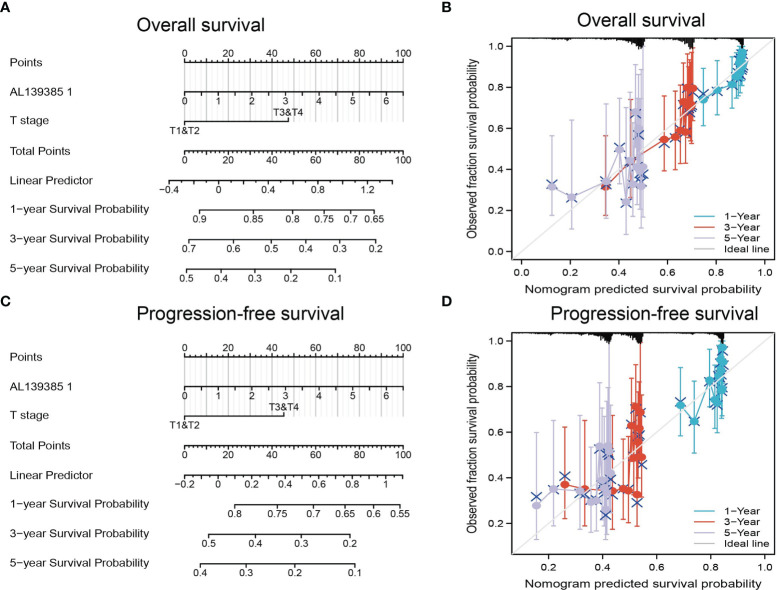
Construction and performance validation of the AL139385.1-based nomogram for lung adenocarcinoma patients. **(A–D)** Nomogram to predict the overall survival and progression-free survival for lung cancer patient, the calibration curve, and Hosmer–Lemeshow test of nomograms in the TCGA lung adenocarcinoma cohort for overall survival and progression-free survival.

The multivariate analysis result confirmed that AL139385.1 is an independent prognostic factor in LUAD. We then constructed a prediction model for OS and PFS by integration AL139385.1 expression and T stage. We established a nomogram to integrate AL139385.1 as a LUAD biomarker, and higher total points on the nomogram for OS and PFS, respectively, indicated a worse prognosis **(** **Figures 4A–D** **).** In summary, these results indicated that the nomogram can well predict short- or long-term survival of patients with LUAD.

### AL139385.1-Related Signaling Pathways Enrichment by GSEA

**Figure 5:**
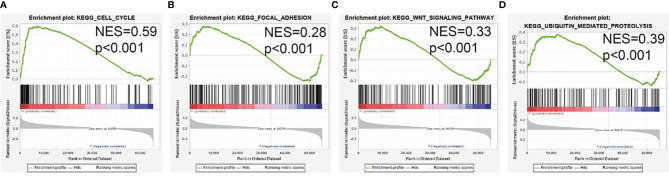
GSEA Identification of AL139385.1-related signaling pathways. **(A–D)** Identification of AL139385.1-related signaling pathways by GSEA software.

To determine the biological function of AL139385.1, we analyzed the DEGs between the low and high AL139385.1 expression groups according to the median expression value of AL139385.1. GSEA pathway analysis result confirmed that AL139385.1 mainly involved in cell cycle, focal adhesion, Wnt signaling pathway, and ubiquitin-mediated proteolysis **(** **Figures 5A–D** **)**.

### Correlation Between AL139385.1 Expression and Immune Infiltration

**Figure 6:**
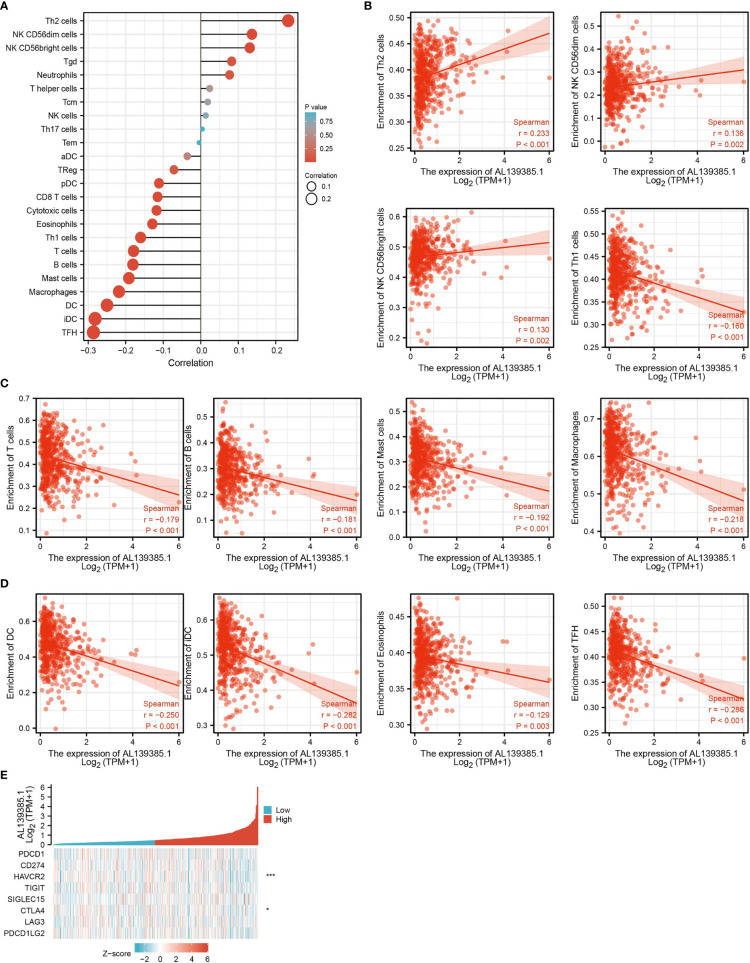
Correlation analysis of AL139385.1 expression and infiltration levels of immune cells in LUAD. **(A–D)**The correlation between AL139385.1 expression and the infiltration levels of 26 immune cells in LUAD. **(E)** The correlation between AL139385.1 expression and immune checkpoint–related genes in LUAD. *P < 0.05, ***P < 0.001.

Infiltration of immune cells has an indispensable role in cancer progression (24), and we then examined the relationship between AL139385.1 expression and the infiltration levels of 24-type immune cells in LUAD using ssGSEA method. We found that AL139385.1 was positively associated with the abundance of Th2, NK CD56DIM, and NK CD56bright cells and negatively correlated with the abundance of Th1 cells, T cells, B cells, Mast cells, macrophages, DC, iDC, and TFH in LUAD **(** **Figures 6A–D** **)**.

Considering that AL139385.1 might be the potential oncogene in LUAD, the relationship of AL139385.1 with PDCD1, CD274, HAVCR2, TIGIT, SIGLEC15, CTLA4, LAG3, and PDCD1LG2 in LUAD was assessed. As a result, we found that the expression levels of AL139385.1 were significant positive correlation with HAVCR2 and CTLA4 in LUAD **(** **Figure 6E** **)**. These results indicated that tumor immune escape and antitumor immunity might be involved in AL139385.1-mediated carcinogenic processes of LUAD.

### AL139385.1-Related miRNA–mRNA Network in LUAD

**Figure 7:**
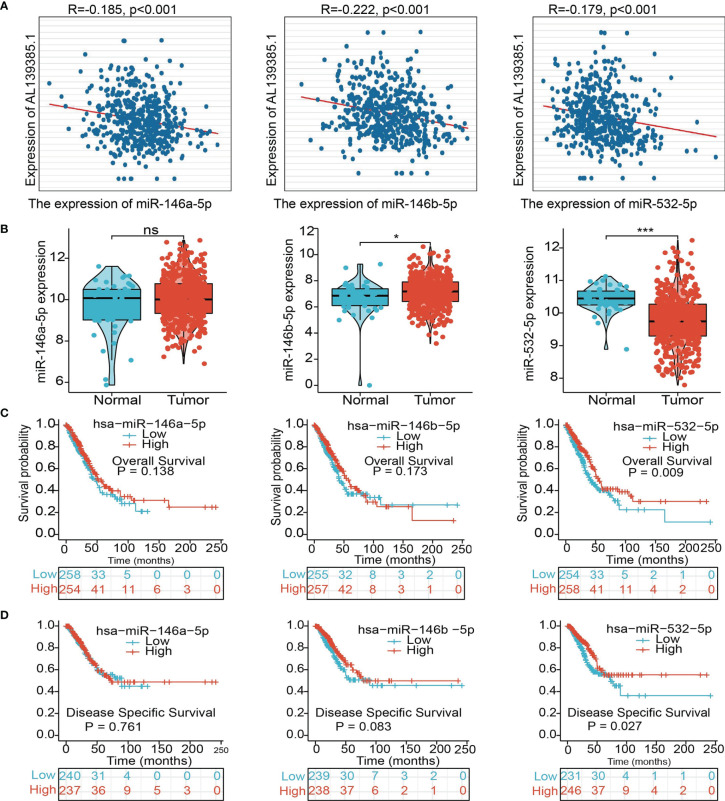
Analysis of the potential miRNAs of AL139385.1. **(A)** Analysis of the correlations between AL139385.1 expression and miR-146a-5p, miR-146b-5p, and miR-532-5p in TCGA-LUAD. **(B)** Analysis of miR-146a-5p, miR-146b-5p, and miR-532-5p expression in lung cancer and adjacent normal tissues in the TCGA database. **(C, D)** Association between miR-146a-5p, miR-146b-5p, and miR-532-5p expression and outcomes of patients with LUAD. NS: P > 0.05,*P < 0.05, and ***P < 0.001.

**Figure 8:**
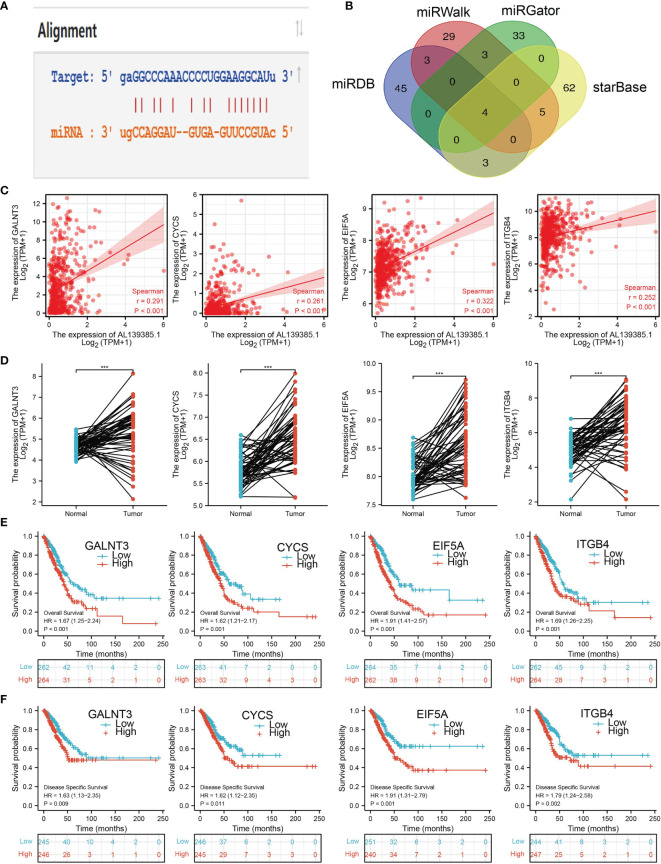
Analysis of the potential mRNAs of AL139385.1/miR-532-5p. **(A)** Sequence match between miR-532-5p and AL139385.1. **(B)** Identifying GALNT3, CYCS, EIF5A, and ITGB4 as the downstream target of miRNA-532-5p using various datasets. **(C)** Correlations between AL139385.1 expression and GALNT3, CYCS, EIF5A, and ITGB4 in LUAD. **(D)** The expression level of GALNT3, CYCS, EIF5A, and ITGB4 in LUAD. **(E, F)** The overall survival and disease-free survival of GALNT3, CYCS, EIF5A, and ITGB4 in LUAD. ***P < 0.001.

To further explore the AL139385.1-mediated downstream regulatory mechanism involved in LUAD progression, we used starBase database to predict the potential miRNAs that bind with AL139385.1; we obtained a total of 27 miRNAs **(** **Supplementary Table 1** **)**. Based on the competitive endogenous RNAs theory, lncRNAs are able to upregulate the mRNA level *via* reducing the expression of miRNAs. Therefore, lncRNA should be positively correlated with mRNA and negatively correlated with miRNA. Among all the 27miRNAs, has-miR-146a-5p, has-miR-146b-5p, and has-miR-532-5p were negatively correlated with AL139385.1 in LUAD **(** **Figure 7A** **)**. We also found that only has-miR-532-5p downregulates in LUAD and correlated with poor prognosis in patients with LUAD. No correlation between prognosis and has-miR-146a-5p and has-miR-146b-5p level was observed **(** **Figures 7B–D** **)**. Therefore, we choose has-miR-532-5p to conduct downstream analysis. Potential binding site between the has-miR-532-5p and AL139385.1 was predicted by starBase **(** **Figure 8A** **)**.

### Identification the Potential Downstream Target of AL139385.1/miR-532-5p in LUAD

**Figure 9:**
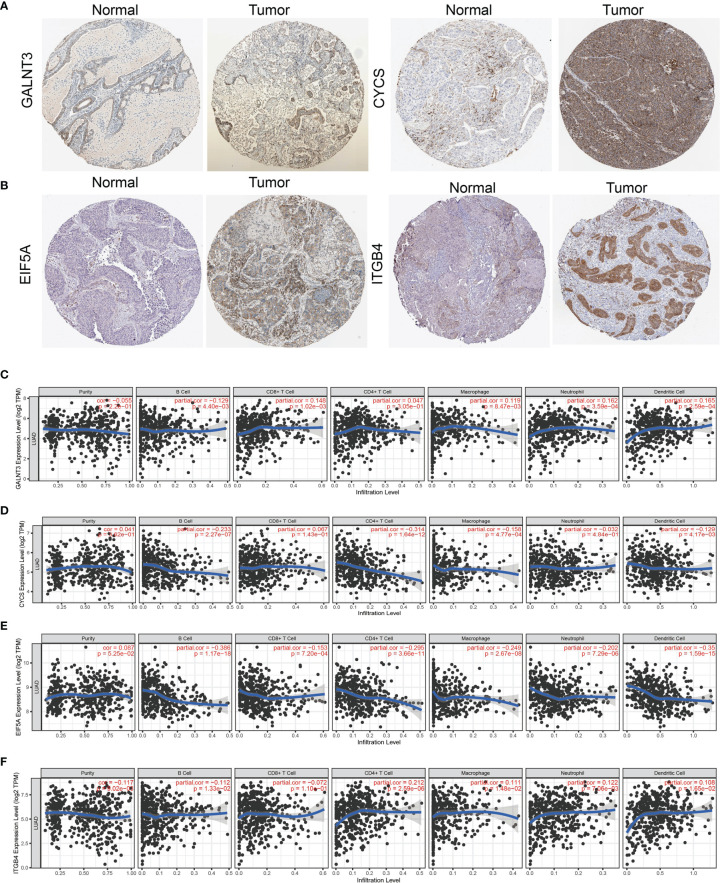
Analysis of the GALNT3, CYCS, EIF5A, and ITGB4 expression and the immune level of correlation. **(A, B)** The protein expression of GALNT3, CYCS, EIF5A, and ITGB4 in LUAD examined by HPA database. **(C–F)** The expression of GALNT3, CYCS, EIF5A, and ITGB4 in LUAD was correlated with tumor purity, B cells, CD4^+^ T cells, CD8^+^ T cells, macrophages, neutrophils, and dendritic cells.

We further investigated the target genes of miR-532-5p that play critical roles in the progression of LUAD. First, we predicted the target in StarBase, miRDB, miRWalk, and miRGator (9, 12–14). According to the prediction of target genes, we found four genes (GALNT3, CYCS, EIF5A, and ITGB4) **(** **Figure 8B** **)**. Importantly, the expression of GALNT3, CYCS, EIF5A, and ITGB4 were positively correlated with that of AL139385.1 in LUAD **(** **Figure 8C** **)**. Furthermore, we employed the TCGA LUAD dataset to explore the expression level and prognosis in LUAD. We found that GALNT3, CYCS, EIF5A, and ITGB4 were significantly upregulated in LUAD and associated with OS and DSS in patients with LUAD **(** **Figures 8D–F** **)**. IHC results obtained from HPA database also confirm that GALNT3, CYCS, EIF5A, and ITGB4 were significantly upregulated in LUAD tissues than normal tissues **(** **Figures 9A, B** **)**.

Finally, we used TIMER database determine the correlations between GALNT3, CYCS, EIF5A, and ITGB4 and six types of tumor-infiltrating immune cells. Results confirmed that GALNT3 expression was negatively correlated with the cell infiltration of B cells, positively associated with the cell infiltration of CD8^+^ T cells, CD4^+^ T cells, macrophage, neutrophils, and dendritic cells **(** **Figure 9C** **)**. CYCS expression was negatively correlated with the cell infiltration of B cells, CD8^+^ T cells, CD4^+^ T cells, macrophage, neutrophils, and dendritic cells **(** **Figure 9D** **)**. EIF5A expression was negatively correlated with six types of tumor-infiltrating immune cells **(** **Figure 9E** **)**. On the contrary, ITGB4 was positively associated with the cell infiltration of CD4^+^ T cells, macrophage, neutrophils, and dendritic cells in LUAD **(** **Figure 9F** **)**.

### AL139385.1 Regulates Proliferation and Migration of LUAD Cells *In Vitro*

**Figure 10:**
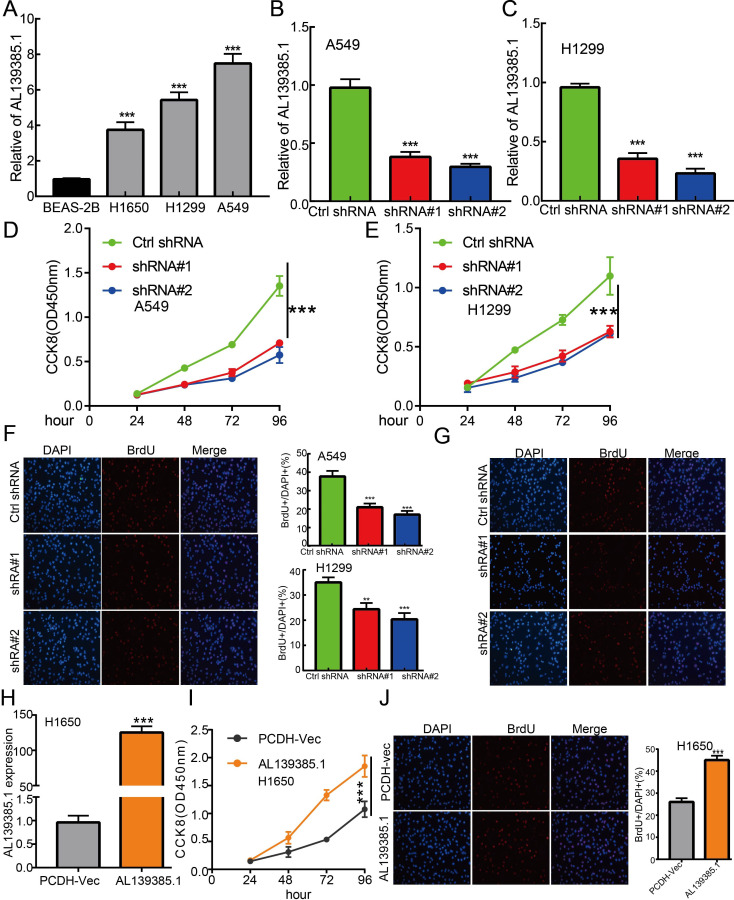
lncRNA-AL139385.1 regulates LUAD cell proliferation *in vitro.* **(A)** The relative expression level of AL139385.1 in lung adenocarcinoma cancerous cell lines, including H1299, H1650, and A549 examined by Real-time RT-PCR, compared to normal human bronchial epithelial cell line: BEAS-2B. **(B, C)** Establishment of AL139385.1 knockdown cell lines in A549 and H1299 verified by Real-time RT-PCR assay. **(D–G)** Knockdown of AL139385.1 significantly inhibits cell proliferation in A549 and H1299 cells, as measured by CCK8 and BrdU incorporation assay. **(H)** Establishment of AL139385.1 overall cell lines in H1650 verified by Real-time RT-PCR assay. **(I, J)** Overexpression of AL139385.1 significantly promotes cell proliferation in H1650 cells, as measured by CCK8 and BrdU incorporation assay. **P < 0.01, ***P < 0.001. shRNA#1=AL139385.1 shRNA#1, shRNA#2= AL139385.1 shRNA#2. AL139385.1=PCDH-AL139385.1.

**Figure 11:**
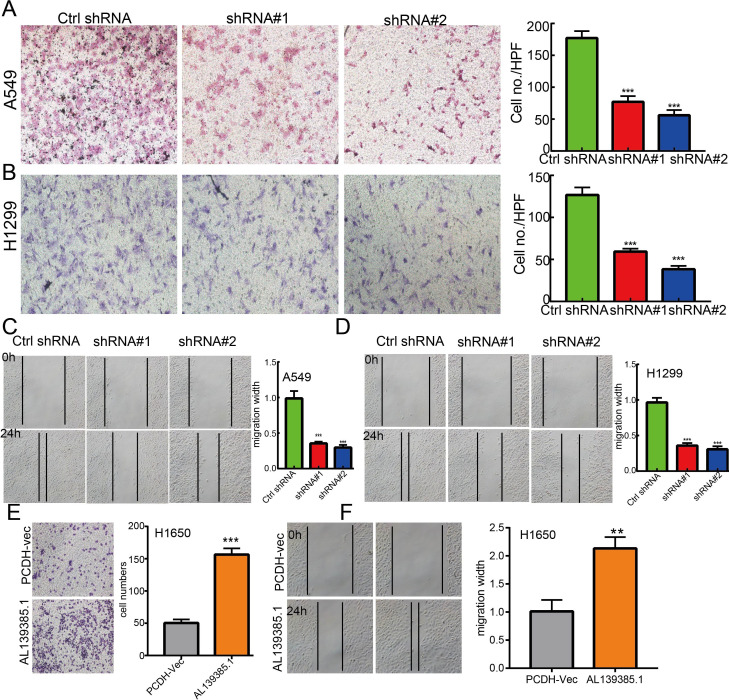
lncRNA-AL139385.1 regulates LUAD cell migration *in vitro*. **(A, B)** Knockdown of lncRNA AL139385.1 dramatically inhibits A549 and H1299 cells migration ability examined by transwell assay. **(C, D)** Knockdown of AL139385.1 dramatically inhibits A549 and H1299 cells migration ability examined by wound healing assay. **(E, F)** Overexpression of AL139385.1 significantly promotes cell migration in H1650 cells, as measured by transwell and wound healing assay. **P < 0.01,***P < 0.001. shRNA#1=AL139385.1 shRNA#1, shRNA#2= AL139385.1 shRNA#2. AL139385.1=PCDH-AL139385.1.

The above studies indicated that AL139385.1 expression was distinctly upregulated in LUAD tissues, and AL139385.1 might influence the progression in LUAD. To further investigate the biological role of AL139385.1 in LUAD, we first confirmed that the expression of AL139385.1 was significantly upregulated in H1650, H1299, and A549 lung cancer cell lines **(** **Figure 10A** **).** Moreover, specific shRNA for AL139385.1 was used to construct A549 and H1299 cells with stable knockdown of AL139385.1 expression. The knockdown efficiencies in transformed cell lines were detected by qRT-PCR analysis **(** **Figures 10B, C** **)**. It was confirmed that knockdown of AL139385.1 reduced the proliferative capacity of A549 and H1299 cells **(** **Figures 10D–G** **)** upon CCK8 and BrdU assays. Moreover, we also established overexpression of AL139385.1 cell lines and used qRT-PCR assay to validate the overexpression efficiency **(** **Figure 10H** **)**. We found that overexpression of AL139385.1 promotes cell proliferation of LUAD **(** **Figures 10I, J** **)**. Moreover, transwell and wound healing assay revealed that the migration abilities of A549 and H1299 cells were significantly inhibited through downregulating AL139385.1 expression level **(** **Figures 11A–D** **)**. On the contrary, overexpression of AL139385.1 promotes cell migration of LUAD **(** **Figures 11E, F** **)**. Together, our data suggest that AL139385.1 is functionally important in regulating cell proliferation and migration of LUAD cells.

## Discussion

Here, we found that AL139385.1 was upregulated in LUAD tissues, and its high expression was correlated with pathological stage, TNM stage, and poor survival ability. We also constructed a putative ceRNA network of AL139385.1 in LUAD by sponging multiple miRNAs and mRNAs. Although some of these findings lack additional experimental verification, our data indicate that AL139385.1 may be a promising target and diagnostic biomarker for LUAD.

Increasing evidence demonstrated the functional and clinical role of lncRNAs involved in the progression (24, 25). For instance, lincRNA OIN1 could act as a tumor oncogenic lincRNA in ovarian cancer, which will be a potential molecular target for treating ovarian cancer (26). Higher lncRNA ANRIL expression was related to increased metastases rates and reduced OS rate in osteosarcoma (27). These studies displayed the vital role of lncRNAs in regulating tumor progressions. This study disclosed that AL139385.1 was upregulated in LUAD tissues and related to crucial clinical characteristics, such as TNM stage, which implied that AL139385.1 expression may be closely associated with tumor progression.

Previous studies indicated that LncRNAs have clinical predictor value in several tumors (28). In the current study, we found that AL139385.1 was highly expressed in LUAD tissues and cell lines. The data also indicated that higher AL139385.1 expression had markedly adverse OS and PFS in patients with LUAD. In addition, ROC curve analysis confirmed that the AUC value of AL139385.1 is 0.808. Results suggested that AL139385.1 may be a promising biomarker for differentiating LUAD tissue from normal lung tissue. These results revealed that AL139385.1 might be a prognosis and diagnostic biomarker in LUAD. We also verified that increased AL139385.1 expression was a significant independent prognostic factor in the TCGA-LUAD cohort that directly correlated with adverse clinical outcomes. We also established a nomogram to integrate AL139385.1 as a LUAD biomarker, and higher total points on the nomogram for OS, and PFS, respectively, indicated a worse prognosis. Furthermore, hypomethylation of the AL139385.1 promoter was associated with its elevated expression in tumor tissues. In *In vitro* assay, we found that AL139385.1 upregulation was mediated by DNA demethylation that promotes lung cancer progression.

Previous studies reported that lncRNA is necessary for the genome stability and cell cycle (29, 30). For example, Gong et al. found that lncRNA JPX was highly expressed in lung cancer metastatic tissues and correlated with tumor size and an advanced stage. Further study showed that JPX promotes LUAD progression *via* activating Wnt/β-catenin signaling (15). In this study, we investigated the underlying mechanisms through which AL139385.1 affected the progression of LUAD. GSEA enrichment confirmed that AL139385.1 was significantly associated with the cell cycle, focal adhesion, Wnt signaling pathway, and ubiquitin-mediated proteolysis.

The lncRNAs could target a series of miRNAs, and the lncRNA-miRNA network reveals a crucial role in tumors (31, 32). For instance, linc00337 expression was increased in LUAD, and linc00337 knockdown could suppress cellular activities *via* targeting miR-1285-3p (33). In this study, the potential AL139385.1-related miRNAs were probed. Among these miRNAs, miR-532-5p was chosen to verify the interplay with AL139385.1. Several studies demonstrated that miR-650 was downregulated in glioma and could inhibited tumor cell proliferation and invasion, as well as a prognostic factor in glioma (34). Moreover, it has been shown that miR-532-5p *via* inhibiting CCR4 suppresses migration and invasion of lung cancer cells (35).

The current results displayed that miR-532-5p is a target miRNA for AL139385.1, which suggests that AL139385.1 might act as a miR-532-5p sponge in LUAD. Thus, we hypothesize that AL139385.1 may promote LUAD progression through targeting miR-532-5p. These findings indicate that AL139385.1 may be a new therapeutic target for the treatment of patients with LUAD. We also utilized various databases to identification the potential targets gene of AL139385.1/miRNA-532-5p in LUAD, including the GALNT3, CYCS, EIF5A, and ITGB4. Subsequent Kaplan–Meier survival analysis suggested that GALNT3, CYCS, EIF5A, and ITGB4, were potential prognostic biomarkers for patients with LUAD. IHC results also confirmed that GALNT3, CYCS, EIF5A, and ITGB4 were highly expressed in lung cancer tissues than normal lung tissues. It has been confirmed that EIF5A was highly expressed in NSCLC and promotes NSCLC cell proliferation and migration (36). Liang et al. found that ITGB4 was significantly increased in LUAD and higher expression level of ITGB4 revealed a worse OS in LUAD (37). Our findings are consistent with previous research studies. In conclusion, this finding provides possible mechanisms underlying the abnormal upregulation of AL139385.1 as well as a comprehensive view of the AL139385.1-mediated ceRNA network in LUAD, thereby highlighting its potential role in diagnosis and therapy. Finally, we found that AL139385.1 was significantly unregulated in NSCLC cells lines, and knockdown of AL139385.1 inhibited cell proliferation and cell migration abilities in LUAD.

This study improves our understanding of the correlation between AL139385.1 and LUAD, but some limitations still exist. First, although we explored the correlation between AL139385.1 and immune infiltration in patients with LUAD, there is lack of experiments to validation the function of AL139385.1 in the tumor microenvironment regulation of LUAD. Second, we uncover that knockdown of AL139385.1 inhibits cell proliferation and cell migration of LUAD. However, the potential molecular mechanisms of AL139385.1 in cancer progression need to be explored in further studies.

## Conclusion

This finding demonstrated that, for the first time, the clinical significances, immune roles, and biological function of AL139385.1 in LUAD. In summary, AL139385.1 is a promising diagnostic and prognostic biomarker for LUAD patients.

## Data Availability Statement

The original contributions presented in the study are included in the article/**Supplementary Material**. Further inquiries can be directed to the corresponding authors.

## Author Contributions

XC, JG, and FZ designed this work and performed related assay, WR, XH, and JP analyzed the data. XN and XJ supervised and wrote the manuscript. All authors have read and approved the final version of the manuscript.

## Funding

This study was supported by Applied Basic Research Project of Yunnan Provincial Science and Technology Department and Kunming Medical University, No.2020001AY070001-117 and the Open Project of The First People’s Hospital of Yunnan Province Clinical Medicine Center (2021LCZXXF‐XZ03).

## Conflict of Interest

The authors declare that the research was conducted in the absence of any commercial or financial relationships that could be construed as a potential conflict of interest.

## Publisher’s Note

All claims expressed in this article are solely those of the authors and do not necessarily represent those of their affiliated organizations, or those of the publisher, the editors and the reviewers. Any product that may be evaluated in this article, or claim that may be made by its manufacturer, is not guaranteed or endorsed by the publisher.
