# Corrigendum: Long non-coding RNA AL139385.1 as a novel prognostic biomarker in lung adenocarcinoma

**DOI:** 10.3389/fonc.2024.1223478

**Published:** 2024-09-03

**Authors:** Xi Chen, Jishu Guo, Fan Zhou, Wenjun Ren, Xiaobin Huang, Jun Pu, Xiaoqun Niu, Xiulin Jiang

**Affiliations:** ^1^ Department of Neurosurgery, The Second Affiliated Hospital of Kunming Medical University, Kunming, China; ^2^ Institute for Ecological Research and Pollution Control of Plateau Lakes, School of Ecology and Environmental Science, Yunnan University, Kunming, China; ^3^ Hematology and Rheumatology Department, The Pu’er People’s Hospital, Pu’er, China; ^4^ Department of Cardiovascular Surgery, The First People’s Hospital of Yunnan Province, Kunming, China; ^5^ Department of Respiratory Medicine, Second Hospital of Kunming Medical University, Kunming, China; ^6^ Kunming College of Life Science, University of Chinese Academy of Sciences, Beijing, China

**Keywords:** AL139385.1, lung adenocarcinoma, prognosis biomarker, DNA methylation, ceRNA, cell proliferation, cell migration

In the published article, there was an error in **Figure 10G** and **Figure 11D** as published. The representative picture of BrdU, Wound healing of H1299 cells was presented incorrectly. The error happened due to duplicate in naming the figures which later was not checked again. The corrected **Figures 10** and **11** and their captions appear below.

**Figure 10:**
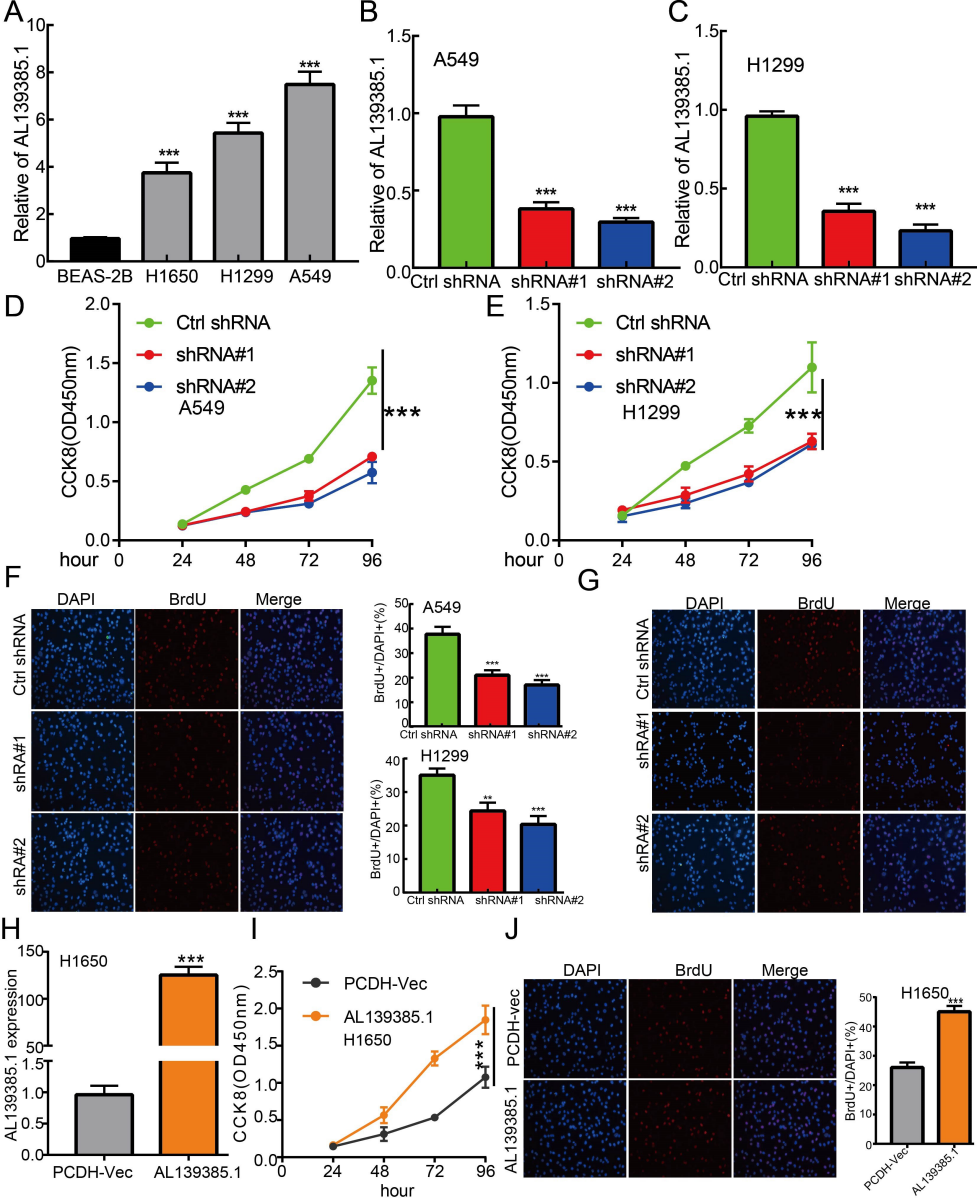
lncRNA-AL139385.1 regulates LUAD cell proliferation *in vitro.* **(A)** The relative expression level of AL139385.1 in lung adenocarcinoma cancerous cell lines, including H1299, H1650 and A549 examined by Real-time RT-PCR, compared to normal human bronchial epithelial cell line: BEAS-2B. **(B, C)** Establishment of AL139385.1 knockdown cell lines in A549 and H1299 verified by Real-time RT-PCR assay **(D-G)** Knockdown of AL139385.1 significantly inhibits cell proliferation in A549 and H1299 cells, as measured by CCK8 and BrdU incorporation assay. **(H)** Establishment of AL139385.1 overall cell lines in H1650 verified by Real-time RT-PCR assay **(I, J)** Overexpression of AL139385.1 significantly promotes cell proliferation in H1650 cells, as measured by CCK8 and BrdU incorporation assay. **P < 0.01, ***P < 0.001. shRNA#1=AL139385.1 shRNA#1, shRNA#2= AL139385.1 shRNA#2. AL139385.1=PCDH-AL139385.1.

**Figure 11:**
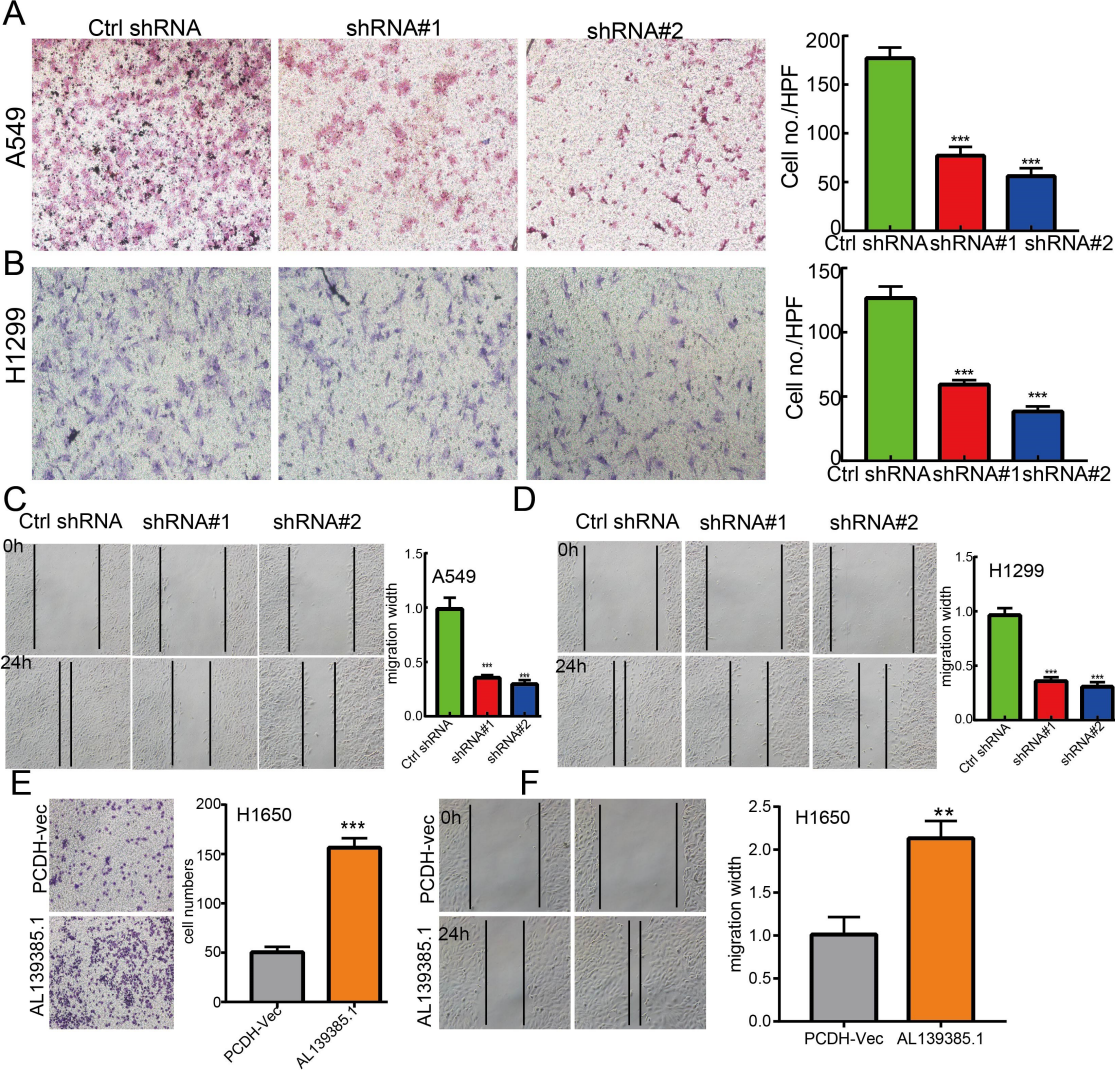
lncRNA-AL139385.1 regulates LUAD cell migration *in vitro.* **(A, B)** Knockdown of lncRNA AL139385.1 dramatically inhibits A549 and H1299 cells migration ability examined by transwell assay. **(C, D)** Knockdown of AL139385.1 dramatically inhibits A549 and H1299 cells migration ability examined by wound healing assay. **(E, F)** Overexpression of AL139385.1 significantly promotes cell migration in H1650 cells, as measured by transwell and wound healing assay. **P < 0.01,***P < 0.001. shRNA#1=AL139385.1 shRNA#1, shRNA#2= AL139385.1 shRNA#2. AL139385.1=PCDH-AL139385.1.

The authors apologize for this error and state that this does not change the scientific conclusions of the article in any way. The original article has been updated.

